# A Rare Event Causing Blindness in an Unrecognised Pregnancy: A Case Report

**DOI:** 10.7759/cureus.115976

**Published:** 2026-09-08

**Authors:** Fang Ying Lim, Norshamsiah Md Din, Nurul Huda Md Amin, Nor Syahira Shariffudin

**Affiliations:** 1 Department of Ophthalmology, Malacca General Hospital, Melaka, MYS; 2 Department of Ophthalmology, Universiti Kebangsaan Malaysia Medical Centre, Kuala Lumpur, MYS

**Keywords:** macular ischemia, optic disc swelling, pre-eclampsia, prenatal care, purtscher-like retinopathy, serous macular detachment

## Abstract

Purtscher-like retinopathy (PLR) is a rare, non-traumatic microembolic angiopathy characteristically associated with systemic endothelial conditions, including pre-eclampsia. While PLR typically exhibits a self-limiting course with a highly favourable visual prognosis, severe cases result in permanent, treatment-refractory blindness. We present a catastrophic, atypical case of bilateral PLR in an unrecognised pregnancy without any antenatal care. A 31-year-old primigravida without prior antenatal screening presented with a three-day history of sudden, bilateral central blurry vision and was newly diagnosed with severe pre-eclampsia. On presentation, her best-corrected visual acuity was 2/60 in the right eye and 3/60 in the left eye. Fundoscopy revealed prominent bilateral optic disc swelling, a complete macular star, and generalised Purtscher flecken. Optical coherence tomography confirmed severe macular oedema accompanied by serous macular detachment. Despite timely intervention with high-dose intravenous methylprednisolone, followed by a three-week tapering course of oral prednisolone, her visual acuity remained poor at the two-month follow-up, after which the patient defaulted. This case demonstrates that the co-existence of PLR with serous macular detachment represents an unusually severe tier of blood-retinal barrier breakdown. Furthermore, it highlights that a total lack of prenatal monitoring allows gestational hypertensive vasospasm to cascade, precipitating irreversible macular ischaemia that is entirely refractory to aggressive rescue corticosteroid therapy.

## Introduction

Purtscher-like retinopathy (PLR) is a distinct, non-traumatic microembolic angiopathy closely associated with systemic conditions such as acute pancreatitis, renal failure, and pre-eclampsia. It happens due to retinal arteriolar embolisation by leukocyte aggregation in response to complement activation [1]. Clinically, it presents as sudden vision loss with pathognomonic "Purtscher flecken" and cotton-wool spots, which are reversible postpartum [2]. Although PLR has been documented sporadically in postpartum patients [3], its emergence in the absence of prior antenatal surveillance underscores the diagnostic challenges in managing severe, unmonitored pre-eclampsia. The pathophysiology in such cases often involves profound endothelial dysfunction and choroidal ischaemia, which can exacerbate the blood-retinal barrier breakdown [4]. Consequently, the resulting retinal arteriolar occlusions and subsequent ischaemia may manifest clinically as either gross macular ischaemia or extensive serous retinal detachment, further complicating the ocular prognosis [5,6].

Herein, we report an unusual and catastrophic case of a 31-year-old peripartum female who presented with profound, irreversible bilateral blindness. This case highlights the critical impact of unmanaged gestational hypertension on the development of irreversible ocular ischaemia. This underscores the necessity of distinguishing such severe, non-resolving manifestations from the more common, transient visual disturbances typically observed in hypertensive disorders of pregnancy [7].

## Case presentation

A 31-year-old Malay primigravida with no prior antenatal screening due to an unrecognised pregnancy and no previous comorbidities presented with a three-day history of sudden, bilateral, central loss of vision occurring before delivery. She sought treatment on the third day of presentation, which was during the active phase of labour at 36 weeks of gestation and was newly diagnosed with severe pre-eclampsia. She had no eye pain, double vision, floaters, flashes, headache, nausea, vomiting, or any other neurological symptoms.

On ophthalmic examination, her best-corrected visual acuity (BCVA) was 2/60 and 3/60 for the right and left eyes, respectively. Anterior segment evaluation was normal bilaterally, and intraocular pressure was 14 mmHg. Fundoscopy revealed bilateral extensive optic disc oedema, macular star, and generalised Purtscher flecken (Figure 1A). The remaining macula and peripheral retina were flat. Her blood pressure on presentation was 215/163, and she had pedal oedema to the mid-shin. The obstetric team had given her oral and intravenous antihypertensive medications together with intravenous magnesium sulphate according to the pre-eclampsia protocol. Her blood pressure improved slowly after treatment initiation.

She had proteinuria with low total protein and albumin levels and elevated alkaline phosphatase (ALP) and lactate dehydrogenase (LDH), but not significant enough to diagnose HELLP (haemolysis, elevated liver enzymes, and low platelet count) syndrome. Her haemoglobin was also low; however, her bilirubin was normal, thus excluding haemolysis (Table 1). Optical coherence tomography (OCT) of the macula showed macular oedema with serous macular detachment (Figure 2A). She was treated with high-dose intravenous methylprednisolone 250 mg four times a day for three days, followed by a three-week tapering course of oral prednisolone. At the two-month follow-up, OCT findings (Figure 2B) and fundus presentation (Figure 1B) showed improvement, but her visual acuity remained unchanged despite treatment. She subsequently defaulted on follow-up after completing treatment due to logistical issues.

**Table 1:** Laboratory investigations on presentation.

Laboratory parameter	Patient's value	Reference range	Status
Haemoglobin	11.0 g/dL	13.0-17.0 g/dL	Low
White blood cells	9.7 10^9/L	4.0-10.0 10^9/L	Normal
Platelets	198 10^9/L	150-410 10^9/L	Normal
Sodium	136 mmol/L	136-145 mmol/L	Normal
Potassium	3.9 mmol/L	3.5-5.1 mmol/L	Normal
Urea	6.7 mmol/L	2.8-8.1 mmol/L	Normal
Creatinine	80 umol/L	44-80 umol/L	Normal
Total protein	58 g/L	64-83 g/L	Low
Total bilirubin	11.4 umol/L	<24 umol/L	Normal
Albumin	27 g/L	35-52 g/L	Low
Globulin	31 g/L	25-39 g/L	Normal
Alanine transaminase (ALT)	9 U/L	10-35 U/L	Low
Alkaline phosphatase ( ALP)	194 U/L	35-104 U/L	High
Aspartate transaminase (AST)	32 U/L	10-35 U/L	Normal
Lactate dehydrogenase	406 U/L	<250 U/L	High
Urine protein	Present		
Urine protein creatinine ratio	249.49 mg/mmol	<15 mg/mmol	Overt proteinuria

**Figure 1:**
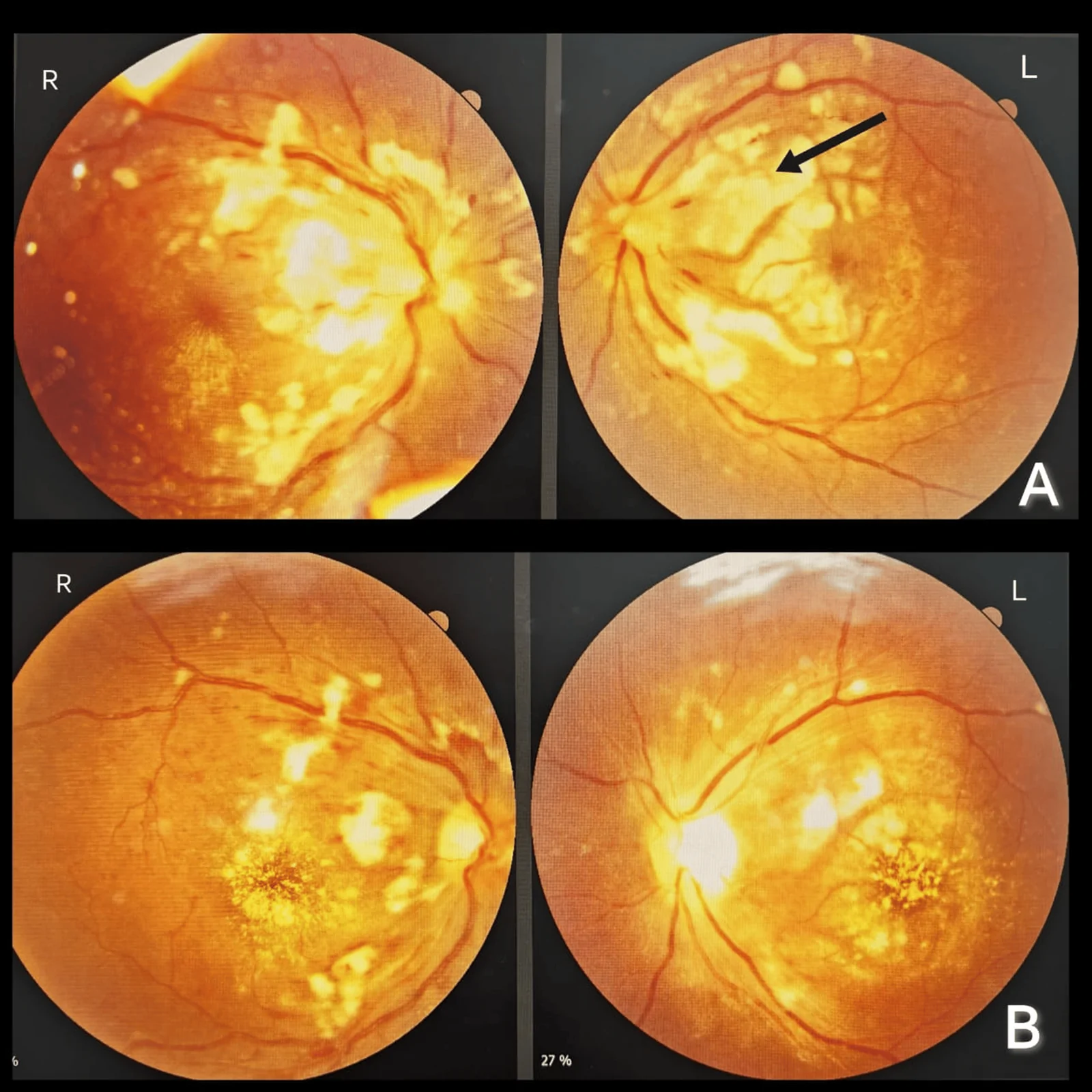
Fundus examination. (A) Fundus examination on presentation showed bilateral extensive optic disc oedema, macular star, and generalised Purtscher flecken. (B) Fundus examination two months later. The black arrow indicates Purtscher flecken.

**Figure 2:**
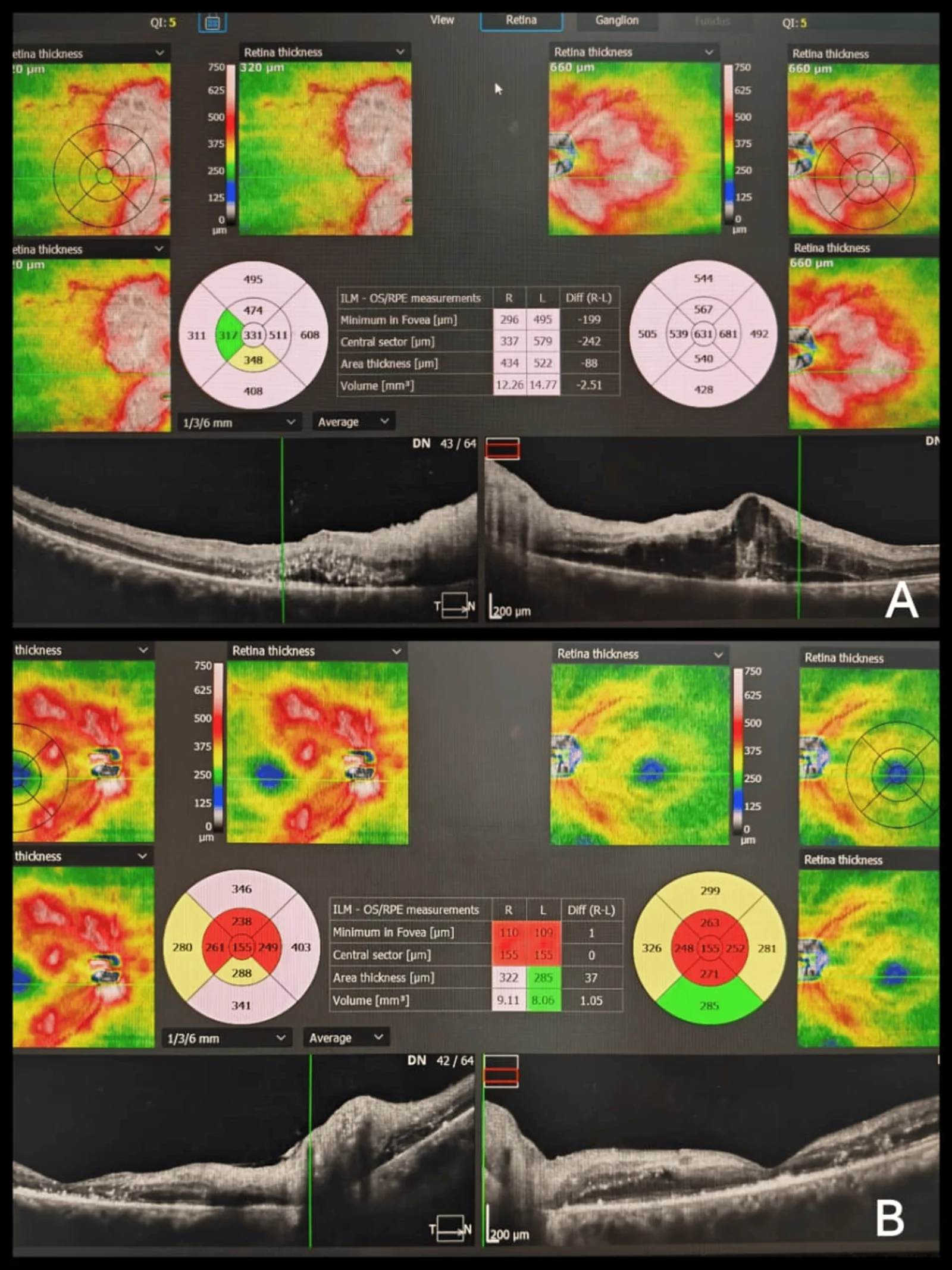
Optical coherence tomography (OCT). (A) OCT on presentation showed macular oedema accompanied by serous macular detachment. (B) OCT at two-month follow-up showed improvement.

## Discussion

Pre-eclampsia is a multi-system disorder affecting pregnancy in the third trimester, which is characterised by new-onset hypertension and proteinuria [8]. The ocular manifestations affect 25% to 40% of patients [9]. They normally resolve spontaneously postpartum. However, in 0.1% to 3% of cases, permanent vision loss can happen [9].

Purtscher retinopathy is an uncommon condition normally associated with trauma [10]. In a non-traumatic condition such as pancreatitis, fat embolism, renal failure, and pre-eclampsia, this condition is described as PLR [11]. PLR can have a variable visual outcome, and the prognosis depends largely on the disease severity at presentation [11]. Some cases may recover spontaneously after five months, while others may have persistent poor vision, as in our case. While typical pre-eclampsia-associated serous retinal detachment usually exhibits favourable anatomical and functional recovery within weeks [12,13], our patient’s irreversible ischaemic damage suggests that underlying microvascular compromise can lead to permanent structural degradation when prenatal intervention is absent. Furthermore, while localised subretinal fluid accumulations in pre-eclamptic patients often regress with blood pressure stabilisation [14,15], widespread arteriolar occlusion in PLR suggests a more severe systemic vascular collapse that may render traditional hypertensive management insufficient to restore retinal function [16].

Currently, no fixed guideline exists for treating PLR [17]. Some case reports show treatment with intravenous steroids, which causes the symptoms to subside, whereas some revealed an improvement in the patient's condition with only observation [18]. In our case, the patient had been given a course of high-dose steroid treatment; unfortunately, her visual disturbance did not resolve at two months. This may be due to severe macular ischaemia at presentation. She had no antenatal screening, which led to the late detection of pre-eclampsia that could have already induced irreversible ischaemic infarction of the retinal cells before the steroids were administered. Given that the clinical features often overlap, precise diagnostic criteria such as the presence of Purtscher flecken, cotton-wool spots, and specific systemic associations are essential to differentiate this condition from other severe hypertensive retinopathies [19]. Advanced imaging modalities, including OCT angiography, provide critical insights by identifying characteristic microvascular occlusions and confirming an ischaemic pathogenesis [20].

## Conclusions

PLR is a visually threatening eye condition that is rarely reported in conjunction with pre-eclampsia. Its clinical course and visual prognosis vary. Thus, ophthalmologists should maintain a high index of suspicion for this rare complication in patients presenting with unmonitored pre-eclampsia, as early recognition remains the only strategy to potentially mitigate long-term visual morbidity.

Future research should clarify the role of complement activation in the pathophysiology of this microvascular damage to determine whether targeted systemic therapies might be more effective than standard corticosteroid protocols. Additionally, large-scale prospective studies are required to establish whether other vasoactive interventions offer superior outcomes over simple observation in cases of severe ischaemic involvement.
